# Jasmonates act positively in adventitious root formation in petunia cuttings

**DOI:** 10.1186/s12870-015-0615-1

**Published:** 2015-09-22

**Authors:** Sandra Lischweski, Anne Muchow, Daniela Guthörl, Bettina Hause

**Affiliations:** Department of Cell and Metabolic Biology, Leibniz Institute of Plant Biochemistry, Weinberg 3, D06120 Halle/Salle, Germany; Present address: Interdisziplinäres Stoffwechsel-Centrum, Charité, Augustenburger Platz 1, D13353 Berlin, Germany; Present address: IDT Biologika GmbH, Am Pharmapark, D06861 Dessau-Roßlau, Germany; Present address: Institute of Plant Biology, University of Zürich, Zollikerstrasse 107, CH-8008 Zürich, Switzerland

**Keywords:** Adventitious root formation, Auxin, Cell wall invertase, Cuttings, Ethylene, Jasmonic acid, JA-isoleucine, *Petunia hybrida*

## Abstract

**Background:**

Petunia is a model to study the process of adventitious root (AR) formation on leafy cuttings. Excision of cuttings leads to a transient increase in jasmonates, which is regarded as an early, transient and critical event for rooting. Here, the role of jasmonates in AR formation on petunia cuttings has been studied by a reverse genetic approach.

**Results:**

To reduce the endogenous levels of jasmonates, transgenic plants were generated expressing a *Petunia hybrida ALLENE OXIDE CYCLASE (PhAOC)-*RNAi construct. The transgenic plants exhibited strongly reduced *PhAOC* transcript and protein levels as well as diminished accumulation of *cis*-12-oxo-phytodienoic acid, jasmonic acid and jasmonoyl-isoleucine after wounding in comparison to wild type and empty vector expressing plants. Reduced levels of endogenous jasmonates resulted in formation of lower numbers of ARs. However, this effect was not accompanied by altered levels of auxin and aminocyclopropane carboxylate (ACC, precursor of ethylene) or by impaired auxin and ethylene-induced gene expression. Neither activity of cell-wall invertases nor accumulation of soluble sugars was altered by jasmonate deficiency.

**Conclusions:**

Diminished numbers of AR in JA-deficient cuttings suggest that jasmonates act as positive regulators of AR formation in petunia wild type. However, wound-induced rise in jasmonate levels in petunia wild type cuttings seems not to be causal for increased auxin and ethylene levels and for sink establishment.

**Electronic supplementary material:**

The online version of this article (doi:10.1186/s12870-015-0615-1) contains supplementary material, which is available to authorized users.

## Background

Adventitious root (AR) formation in leafy stem cuttings is a crucial process for the propagation of many ornamental plant species. Vegetative propagation of plants relies on the ability of shoot cuttings to effectively generate such roots. There is, however, a dramatic variation between species in their capability to form AR [1]. During AR formation roots develop from non-root tissue, mostly aerial plant parts such as hypocotyls, leaves and stems [2]. The origin of ARs varies among plant species and organ or tissue they develop from [3]. ARs always develop from cells neighboring vascular tissues and can initiate from hypocotyl pericycle cells, phloem or xylem parenchyma cells, young secondary phloem cells, or interfascicular cambium cells. In *Arabidopsis thaliana*, a model often used to investigate AR formation, rooting is induced by pre-etiolation of intact seedlings. After supply with sugars or hormones or after transfer into light they develop roots at the intact hypocotyl [4–7]. In this model system, AR formation occurs without stresses that disrupt root-shoot correlative influences [4]. This is in contrast to leafy cuttings, which are subjected to severance from the donor plant accompanied by injury and the isolation from functional integrity of the whole plant conditions [8]. In both, pre-etiolated seedlings and leafy cuttings, AR formation is a complex process influenced by multiple endogenous and exogenous factors, including phytohormones, light, wounding, and stress [2]. Among the endogenous factors, the phytohormones are the most important modulators of AR formation—it has become obvious that auxin and ethylene (ET) play a central role, but they interact with one another; with other phytohormones and with environmental cues in complex networks [3].

For *Petunia hybrida*, a model plant for AR development on leafy cuttings, a three-phase mechanism was postulated for the metabolic responses involved in AR formation [9] consisting of (i) establishment of a sink, (ii) a recovery phase and of (iii) a maintenance phase. In the first phase, the excision of cuttings leads to rapid and transient increase in the wound-phytohormone jasmonic acid (JA) and a continuous accumulation of soluble and insoluble carbohydrates [9]. It was hypothesized that wounding accompanied by the rise in JA initiates the establishment of a sink tissue necessary to facilitate subsequent AR formation. With that, wound-induced JA accumulation at the cutting stem base has been regarded as an early, transient and critical event for rooting of *Petunia* cuttings. Remarkably, in Arabidopsis intact hypocotyls jasmonates negatively regulate adventitious rooting, and their homeostasis is under control of auxin [10].

Jasmonates are ubiquitously occurring signaling compounds in plants and accumulate in response to biotic and abiotic stress as well as in development [11]. JA and its molecularly active metabolite (+)-*7-iso*-jasmonoyl isoleucine (JA-Ile) are lipid-derived compounds and are synthesized from α-linolenic acid by one of seven different branches of the lipoxygenase (LOX) pathway [12, 13]. LOX and the two following enzymatic steps are located in the plastids and involve the action of an ALLENE OXIDE SYNTHASE and an ALLENE OXIDE CYCLASE (AOC) leading to formation of the intermediate *cis*-12-oxo-phytodienoic acid (OPDA). Further reactions occur in peroxisomes and form JA, which is enzymatically converted to JA-Ile within the cytosol. JA-Ile was demonstrated to mediate binding of the co-receptor proteins CORONATINE INSENSITIVE1 (COI1) and JASMONATE ZIM DOMAIN (JAZ), thereby triggering JA responses [14–17].

Within the JA pathway the AOC-catalyzed step is regarded as the crucial step as here the exclusive formation of the enantiomeric form occurring in natural cyclopentanones like JA and JA-Ile is facilitated [18, 19]. In petunia, the AOC is constitutively present in stem tissue, and has constantly high activity [9]. Additionally, *PhAOC* belongs to the wound-induced genes, and its transcript levels increase following endogenous rise in JA/JA-Ile [9].

To study the role of JA in AR formation in leafy cuttings of petunia by a reverse genetic approach, the cDNA of *PhAOC* was used to generate an RNAi construct for the stable transformation of plants to partially suppress *PhAOC* expression. Our data clearly show that this suppression markedly decreases the accumulation of jasmonates as tested in leaves after mechanical wounding. Additionally, suppression of *PhAOC* caused a delay in AR formation. To get insights into how diminished JA levels might affect AR formation, stem bases of cuttings from transgenic plants were used to comparatively analyze levels of IAA and aminocyclopropane carboxylate (ACC, precursor of ET), but also transcript levels of a cell wall invertase (CWI) encoding gene as well as sugar contents.

## Results and discussion

Among the lipid-derived compounds, octadecanoids and jasmonates have a crucial role in plant responses to biotic and abiotic stresses, but also in developmental processes [20]. JA was suggested to be one of several endogenous factors regulating the formation of AR [4]. For petunia cuttings, their excision is characterized by a fast and transient increase in the level of JA followed by induction of genes encoding JA biosynthesis genes and proteins involved in sink establishment, such as cell wall invertases (CWIs) [9, 21].

### Generation of transgenic plants with reduced jasmonate levels

**Fig. 1:**
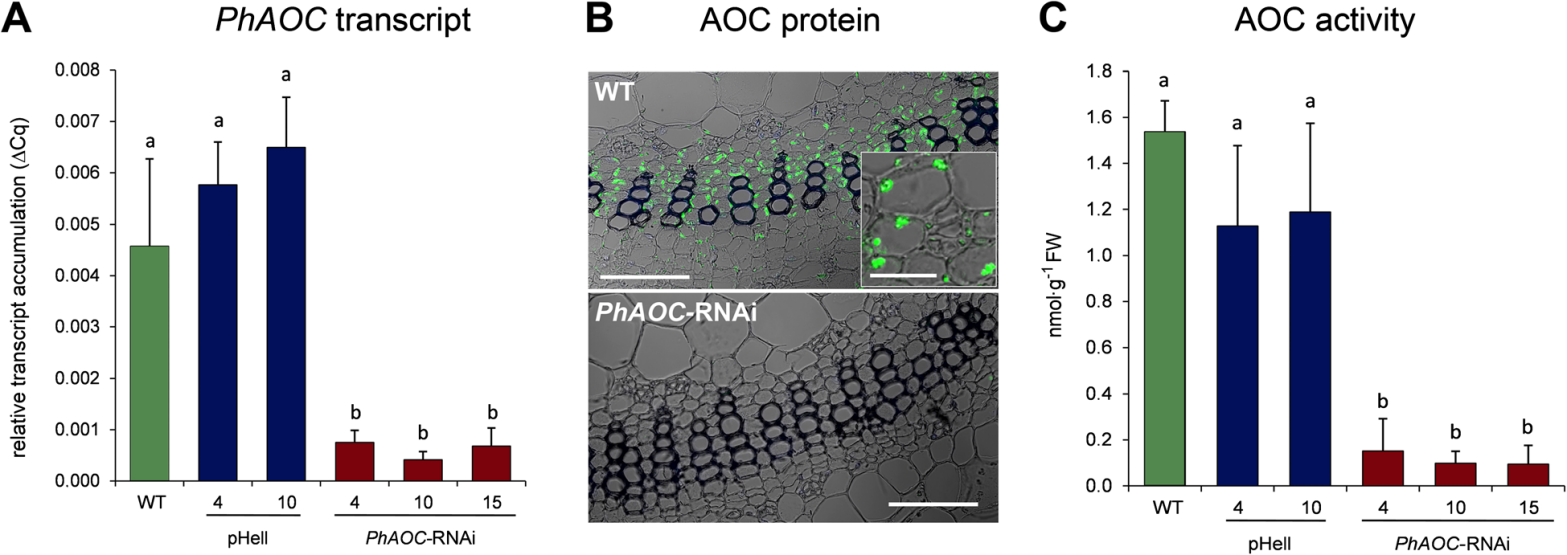
Accumulation of *PhAOC* transcripts, AOC protein and activity in stem bases of cuttings of *Petunia hybrida* expressing *PhAOC*-RNAi. (**a**) Stem bases were collected from leafy cuttings of wild type (WT, green), two lines expressing the empty vector (pHell, blue) and three lines expressing a *PhAOC*-RNAi construct (red) at one hour after excision. Relative *PhAOC* transcript accumulation shown as 2^−ΔCt^ values in relation to the reference gene *PhRSP13* was determined by qRT-PCR. (**b**) AOC protein was detected immunocytologically in cross sections of stem bases using an antibody specific binding to AOC. Note the occurrence of AOC in plastids visible as distinct dots within cells (inset in WT). Bars represent 100 μm and 25 μm in overviews and inset, respectively. (**c**) AOC enzyme activity is given as nmol g^−1^ FW of enzymatically formed OPDA. Each value is represented by the mean of eight independent biological replicates ± SE. Different letters within each graph designate statistically different values (one-way-ANOVA with Tukey’s HSD test, *P* < 0.05)

To elucidate the role of jasmonates in formation of AR in petunia, the endogenous levels of jasmonates were reduced by a transgenic approach. Transgenic plants exhibiting an impaired JA biosynthesis were generated by the RNAi-mediated knock-down of the expression of the gene encoding AOC. AOC of petunia is encoded by a single copy gene (Additional file 1: Figure S1). A 180-bp fragment covering a middle part of the *PhAOC*-coding region was used to generate the *PhAOC*-RNAi construct, which was introduced into petunia plants via *A. tumefaciens* mediated transformation. The expression of *PhAOC-RNAi* is under control of the cauliflower mosaic virus 35S promoter, thereby conferring constitutive expression in all plant tissues. T1 plants were grown together with wild-type plants and plants expressing the empty vector *pHellsgate* (pHell) as controls. Three transgenic *PhAOC*–RNAi lines exhibiting significant reduction of *PhAOC* transcripts (Fig. 1a) were selected. The *PhAOC*–RNAi lines 4, 10 and 15 exhibited residual *PhAOC* transcript levels between 9 and 17 % in comparison to the controls. This reduced level of *PhAOC*-transcript caused a reduction of AOC protein contents to undetectable amounts as shown by immunoblot analyses (Additional file 1: Figure S2). As visualized by the immuno cytological analysis, in wild type stems AOC is located within plastids of internal phloem cells as well as of xylem parenchyma cells of the amphiphloic siphonostele (Fig. 1b, upper micrograph).I In the *PhAOC*–RNAi line, however, the protein amount is below the detection limit (Fig. 1b, lower micrograph). Consequently, cuttings of the *PhAOC*-RNAi lines exhibited a significant reduction of AOC-activity to 9–18 % in comparison to the controls (Fig. 1c).

**Fig. 2:**
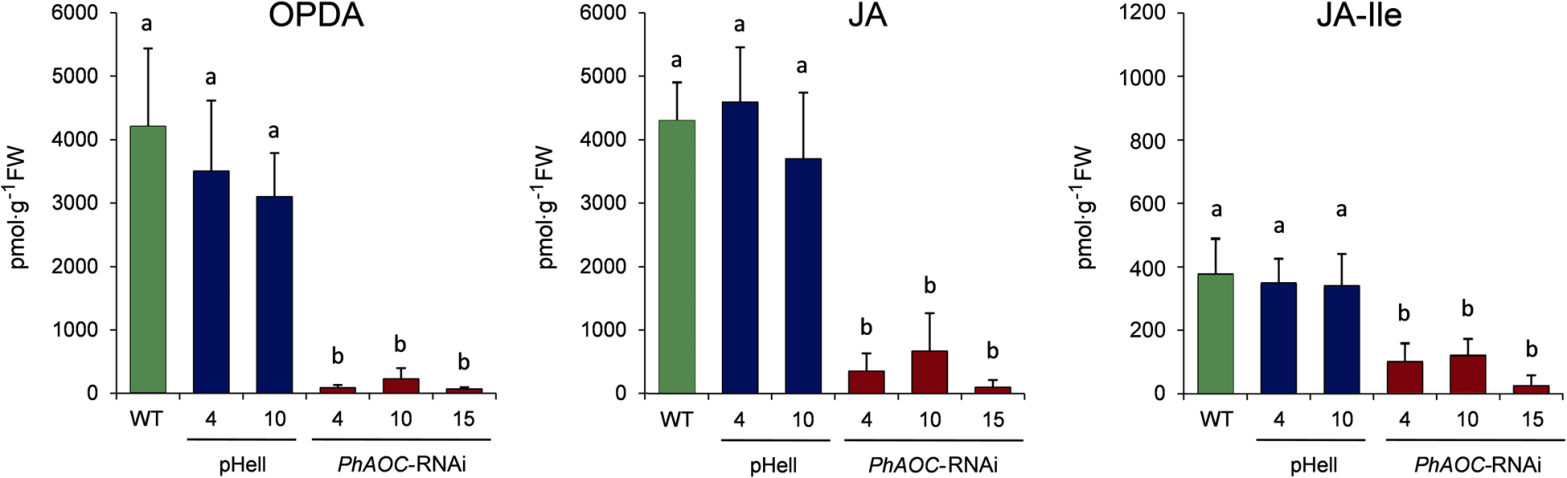
Contents of jasmonates in wounded leaves of *Petunia hybrida* expressing *PhAOC*-RNAi. Contents of 12-oxophytodienoic acid (OPDA), jasmonic acid (JA) and JA-Ile (all given as pmol g^−1^ FW) in leaves of wild type (WT, green), two lines expressing the empty vector (pHell, blue) and three lines expressing a *PhAOC*-RNAi construct (red) harvested one hour after mechanical wounding. Each value is represented by the mean of eight independent biological replicates ± SE. Different letters within each graph designate statistically different values (one-way-ANOVA with Tukey’s HSD test, *P* < 0.05)

Mechanical wounding is a well-known trigger to induce JA biosynthesis [20] and leads also in petunia to a transient rise in jasmonates [9]. In order to check whether the down-regulation of *PhAOC* transcript levels in the transgenic plants was sufficient to modulate endogenous jasmonate levels, contents of OPDA, JA and JA-Ile were determined in leaves one hour after mechanical wounding. Indeed, wound induced levels of OPDA, JA and JA-Ile were significantly lower in *PhAOC*-RNAi lines than in the controls (Fig. 2). Most importantly, the levels of the bioactive jasmonate, JA-Ile, were diminished by a factor of four. All these data indicate that the successful suppression of *PhAOC* expression in transgenic plants is accompanied by a reduced accumulation of jasmonates upon wound stress occurring in stem bases after excision of cuttings.

### Formation of adventitious roots in plants with reduced jasmonate levels

**Fig. 3:**
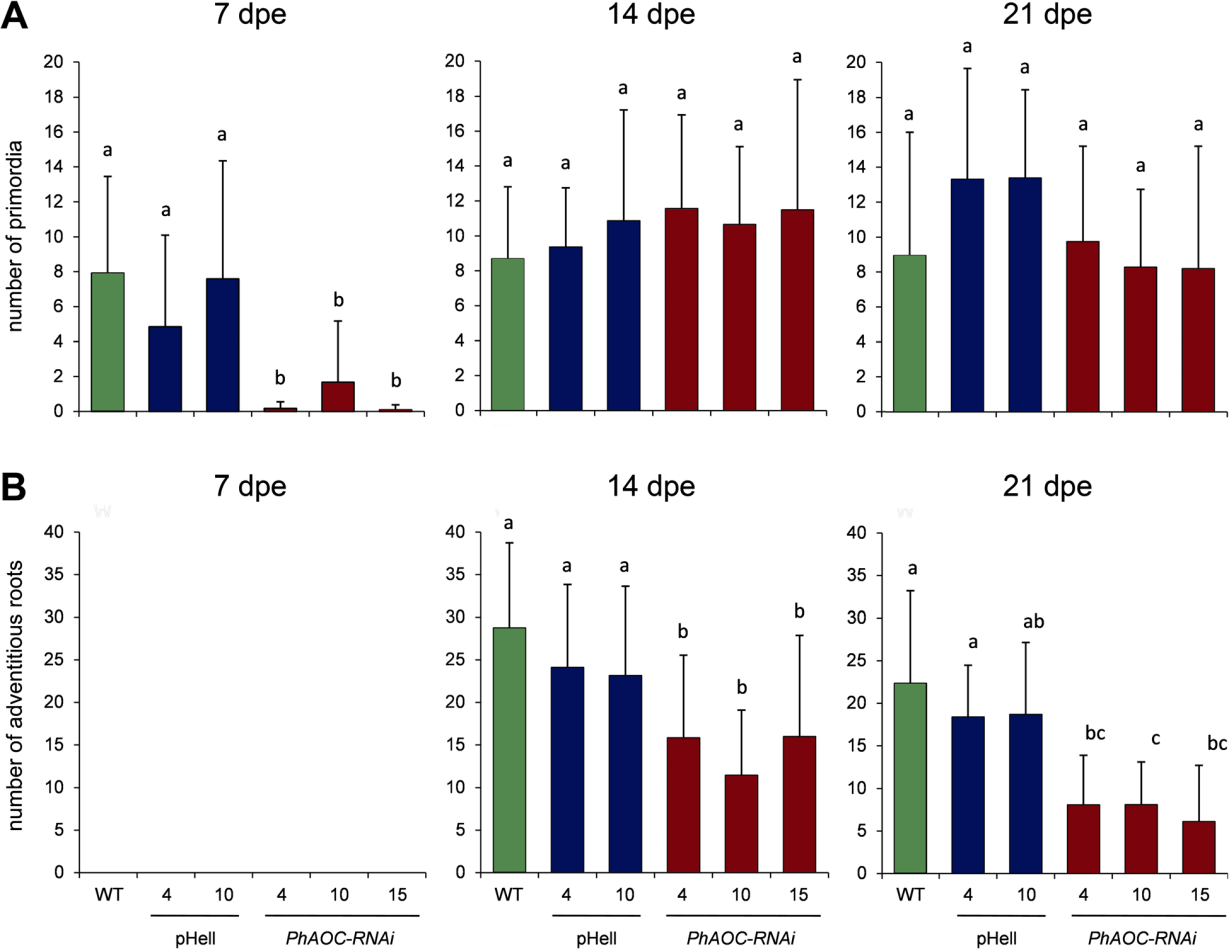
Effect of down-regulation of *PhAOC* on rooting of *Petunia hybrida* cuttings. Rooting was rated at 7, 14 and 21 days post excision (dpe) by counting primordia (**a**) and emerged roots (**b**) in cuttings of wild type (WT, green), two lines expressing the empty vector (pHell, blue) and three lines expressing a *PhAOC*-RNAi construct (red). Each value is represented by the mean of eight independent biological replicates (± SE) consisting each of three technical replicates (single cuttings). Different letters within each graph designate statistically different values (one-way-ANOVA with Tukey’s HSD test, *P* < 0.05)

Using the transgenic lines described above, the question was addressed whether adventitious root development is affected by the reduced levels of jasmonates. For this purpose, leafy cuttings of all plant lines were transferred without any external additives to Perlite as neutral substrate. After 7, 14, and 21 days after excision (dpe) numbers of root primordia and emerged adventitious roots were determined (Fig. 3). Adventitious roots were not yet emerging from stem bases of all lines analyzed at 7 dpe. However, significantly fewer root primordia were detected in the stem base of cuttings of *PhAOC*-RNAi plants in comparison to wild type and the empty vector control plants (Fig. 3a). Numbers of primordia of *PhAOC*-RNAi lines were reduced by 70–90 %. This effect on root primordia was not detectable anymore at the two later time points analyzed. At 14 dpe, adventitious roots emerged from all cuttings (Fig. 3b). Here, cuttings of *PhAOC*-RNAi plants developed significantly fewer roots than cuttings of the controls: Root numbers of cuttings of *PhAOC*-RNAi line 4 and 15 were reduced to 70 %, whereas the root number of cuttings of the *PhAOC*-RNAi line 10 was reduced to 50 % of that of the controls. The reduced number of roots emerged from cuttings of *PhAOC*-RNAi plants was still visible at 21 dpe, although to a lesser extent (Fig. 3). These data suggest that AR formation is delayed in cuttings from plants exhibiting decreased JA levels resulting in a decreased number of roots at 14 and 21 dpe.

These data show that JA deficiency resulted in lower numbers of ARs, but the AR formation itself was not abolished. Therefore, wound-induced jasmonates might function as an accelerator of AR formation in petunia and might act therefore as positive regulators. This is in line with the proposed role of JA in AR formation in petunia and in other species rooted under *in vitro* conditions. For potato (*Solanum tuberosum*) [22] and pea (*Pisum sativum*) [23] stem cuttings, and tobacco (*Nicotiana tabacum*) thin cell layers [24] it was shown that in the presence of exogenous auxin, JA promotes synergistically AR formation. However, these data contrast with data obtained on AR formation in Arabidopsis intact hypocotyls, where JA inhibits AR initiation through the COI1 signaling pathway [10]. These apparent contradictions suggest that there might be either species-specific differences between Arabidopsis and other dicots in respect to AR formation or organ and cultivation-specific differences that may alter the balance between auxin and JA regulating AR formation [3]. Indeed, intact seedlings, de-rooted older plants and cuttings show significant differences, not only in root founding tissues, but also in auxin requirements, sensitivity, and rooting mutant phenotypes [25]. This holds also true for de-rooted seedlings of petunia, which showed reduced numbers of ARs after treatment with jasmonates (Additional file 2: Table S1). Treatments with OPDA, JA and JA-Ile at lower concentrations (0.1–1.0 μM) did not changed AR numbers. Higher concentrations (10 μM) of all compounds, however, significantly reduced the root number, whereas 100 μM JA even completely inhibited AR formation. In contrast to jasmonate treatments, de-rooted seedlings treated with the auxin 2,4-dichlorophenoxy acetic acid (2,4-D) or ACC developed significantly more ARs than control plants (Additional file 2: Table S1). Combined application of JA and either 2,4-D or ACC showed that both, 2.4-D and ACC, suppressed the inhibitory effect of JA on development of ARs, and JA did not diminished the promoting effects of 2.4-D and ACC (Additional file 2: Table S2).

### Effects of diminished jasmonate levels on role of auxin and ethylene in root formation

**Fig. 4:**
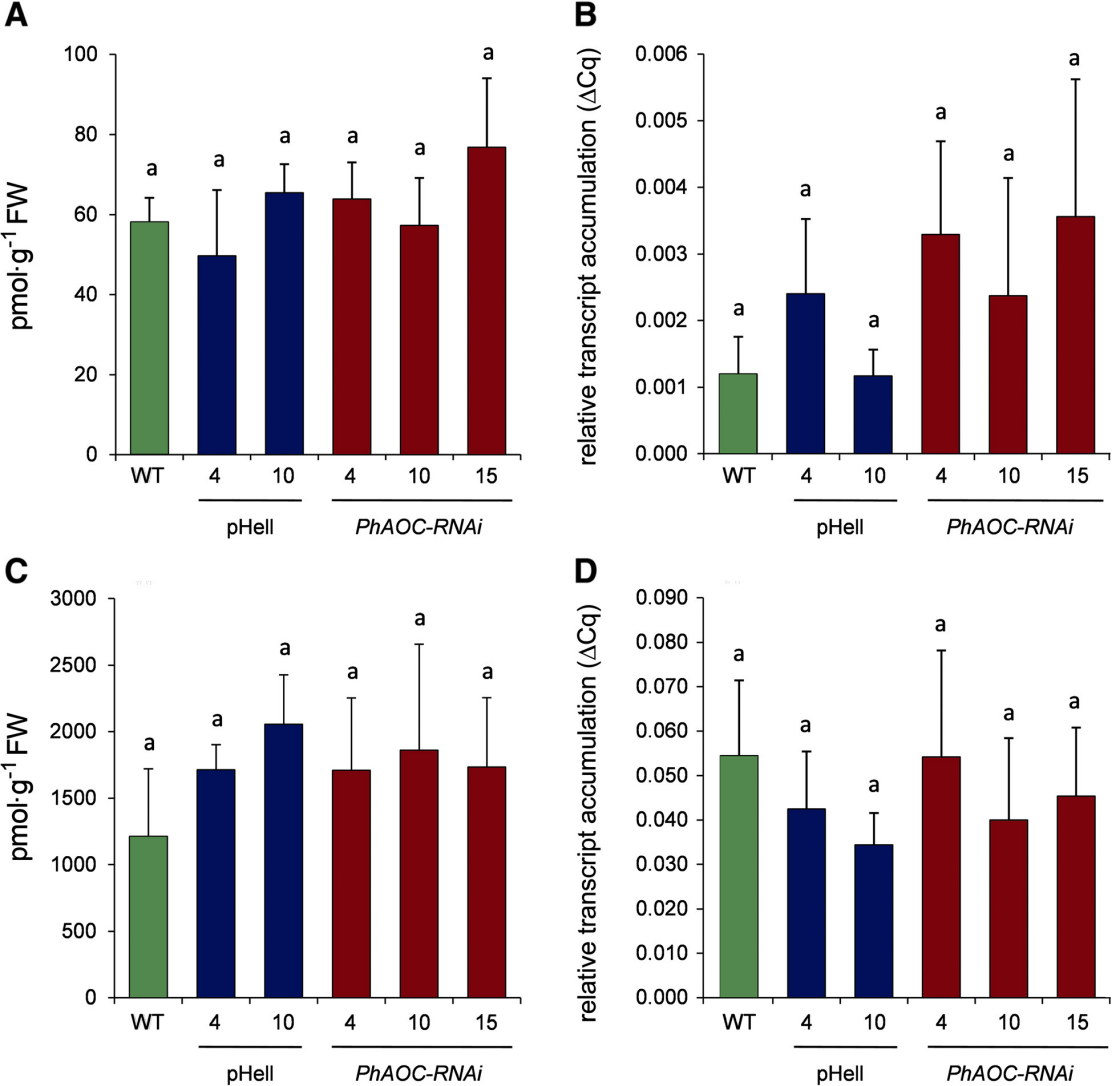
Effect of down-regulation of *PhAOC* on accumulation of indole acetic acid (IAA), amino cyclopropane-1-carboxy acid (ACC) as well as *PhDevA-20-C01* and *PhACO1* transcripts in stem bases of *Petunia hybrida* cuttings. (**a**) content of IAA and (**c**) of ACC, both given as pmol g^−1^ FW at 24 hpe, (**b**) relative *PhDevA-20-C01* transcript accumulation and (**d**) relative *PhACO1* transcript accumulation, both shown as 2^-ΔCt^ values in relation to the reference gene *PhRSP13* at 48 hpe. Stem bases were collected from cuttings of wild type (WT, green), two lines expression the empty vector (pHell, blue) and three lines expressing a *PhAOC*-RNAi construct (red). Each value is represented by the mean of eight independent biological replicates ± SE. Different letters within each graph designate statistically different values (one-way-ANOVA with Tukey’s HSD test, *P* < 0.05)

It is well accepted that ET and auxins play an important role in stimulating the process of AR formation through the different phases [4]. Therefore, the levels of IAA and ACC, the precursor of ET, were determined in JA-deficient cuttings. In parallel, auxin-induced and ET-induced gene expression was monitored by determination of transcript levels of a petunia *GH3* homologue (*PhDevA-20-C01*) and *ACC oxidase1* (*PhACO1*), respectively. In wild type cuttings, free IAA levels increased transiently about two-fold at 2 and 24 hpe followed by increased transcript accumulation of *PhDevA-20-C01* (Additional file 1: Figure S3), whereas ACC levels increased about 200-fold within 24 hpe [21]. Therefore, free IAA and ACC levels as well as transcript accumulation of *PhDevA-20-C01* and *PhACO1* were measured in stem bases of cuttings at 24 and 48 hpe, respectively (Fig. 4). There were no differences in free IAA content and *PhDevA-20-C01* transcript levels between *PhAOC*-RNAi lines and the controls (Fig. 4a, b). Similar results were obtained in respect to ACC levels and transcript accumulation of the ET responsive gene *PhACO1* (Fig. 4c, d), which both also did not show significant differences between JA-deficient plants and wild type/transformation control.

These results showed that diminished JA levels did not change the levels and action of both, auxin and ET in cuttings, although auxin, ET, and JA were found to be coordinately (cooperatively or antagonistically) regulated or exhibit opposite effects on many plant responses [20]. Auxin is considered as inductor of AR formation in leafy cuttings – after detachment of the shoot, basipetal polar transport of auxin contributes to auxin accumulation in the stem base [26]. The rise of free auxin in the basal stem very probably contributes to the early events of AR formation [1, 21, 27, 28]. Additionally, AR formation is also dependent on the action of ET [29, 30], which is synthesized after wounding during the cutting process [27]. It is tempting to speculate that in the process of AR formation JA acts independently from auxin and ET, since diminished JA levels in the transgenic cuttings delayed the formation of AR without changing auxin and ET levels and signaling. It cannot completely be excluded that alterations in hormone levels and related gene expression might occur at other time points than those analyzed. This is, however, not very probable, since the time points used represent in each case the time point of the transient maximum of the respective hormone/transcript level in wild type cuttings (Additional file 1: Figure S3 and [21]).

### Effects of diminished jasmonate levels on cell wall invertase and sugar levels during root formation

CWI is a key enzyme of the apoplastic phloem unloading of transported sucrose and links jasmonate action with primary metabolism [31]. It can establish a sink function of a certain tissue and thus provide a mechanism for flexible and appropriate adjustment to a wide range of internal and external stimuli [32]. To test, whether altered jasmonate levels might affect the induction of the gene encoding CWI and thereby influencing the establishment of the sink [9], transcript levels of *3CL9414* and CWI activity were determined at 4 hpe and 6 hpe, respectively (Table 1). Both time points represent the respective maximum after excision of wild type cuttings [9]. Comparing cutting stem bases of *PhAOC*-RNAi with that of wild type and empty vectors controls, there were no differences in *3CL9414* transcript levels and CWI activity. Additionally, the levels of glucose, fructose and sucrose were determined. The levels of these three compounds reached maximum levels in wild type cuttings at 192 hpe, the time point at which first differentiating root primordia are detectable within the stem base [9]. At this time point the significant reduction in the number of root primordia was found for *PhAOC*-RNAi cuttings in comparison to the controls (Fig. 3). Neither glucose and fructose, nor sucrose exhibited significantly different levels in stem bases of the three genotypes under analysis at 192 hpi (Table 1).

**Table 1:** Transcript accumulation and activity of cell wall invertase, and sugar content in stem bases of cuttings of wild type, pHell and *PhAOC*-RNAi plants

parameter	unit	WT	pHell		*PhAOC*-RNAi		
			line 4	line 10	line 4	line 10	line 15
*3CL9414-*transcript^a^	2^−∆Ct^	20.9 ± 10.1	19.4 ± 7.5	23.1 ± 3.0	19.2 ± 4.6	14.6 ± 7.2	17.8 ± 5.4
invertase activity^b^	pkat/mg protein	12.6 ± 3.7	14.1 ± 3.3	10.2 ± 5.2	10.1 ± 4.2	9.7 ± 3.9	8.3 ± 4.4
glucose^c^	μmol/g f.w.	0.2 ± 0.1	0.2 ± 0.0	0.2 ± 0.1	0.2 ± 0.1	0.2 ± 0.1	0.1 ± 0.1
fructose^c^	μmol/g f.w.	0.4 ± 0.3	0.2 ± 0.1	0.1 ± 0.0	0.5 ± 0.4	0.4 ± 0.4	0.5 ± 0.3
sucrose^c^	μmol/g f.w.	1.8 ± 0.6	1.6 ± 0.2	1.1 ± 0.6	2.7 ± 0.9	2.3 ± 0.7	2.4 ± 1.2

All data are given as mean values ± SD from eight independent, biological replicates. Data were compared by one-way-ANOVA and did not show significantly different values according to Tukey’s HSD test

^a^Transcript data are relative to *PhRSP13* determined at 4 hpe

^b^Cell wall invertase activity determined at 6 hpe

^c^Sugar levels determined at 192 hpe

These results indicate that the wound-induced rise in jasmonates might not be causal for the sink establishment, which was postulated as necessary step in induction of AR formation in petunia [9] and pea [23]. For the onset of AR formation in petunia cuttings, a ‘sink establishment phase’ was defined that might be controlled by wound-induced rise of JA leading in turn to the induction of genes coding for enzymes that degrade sucrose [9]. A similar correlation was drawn from analysis of pea cuttings derived from plants at different developmental stages [23]. In comparison to cuttings from juvenile plants, cuttings from older plants exhibited a postponed accumulation of JA and an impaired AR formation, which was supposed to be caused by a delay in sink-establishment [23]. The results shown here by the transgenic approach, however, contrast to both scenarios and demonstrate that diminished JA biosynthesis did not result in altered carbohydrate levels in petunia cuttings.

## Conclusion

Here, it was demonstrated that the transgenic expression of a *PhAOC*-RNAi construct leads to severe down-regulation of JA biosynthesis in petunia cuttings accompanied by an altered AR formation. The reduced numbers of root primordia and AR on leafy cuttings of *PhAOC*-RNAi plants in comparison to wild type and the empty vector control support the assumption that jasmonates act as positive regulators of AR formation in leafy cuttings of petunia. Since the AR formation was not completely abolished but rather delayed, jasmonates might act as an accelerator of AR formation. In addition, it is unlikely that levels and signaling of auxin and ET as well as gene expression and activity of CWI were altered by JA deficiency and are causal for the delay in AR formation in these plants. Analysis of other phytohormones known to be involved in AR formation, such as cytokinins or strigolactones [2], will be required to unravel how jasmonates regulate AR formation.

## Methods

### Plant material and rooting experiments

*Petunia hybrida* cv. Mitchell was grown as described recently [33]. Plants were cultivated in growth-chambers (temperature 22 °C, humidity 60 %, 10 h light per day), watered with tap water, and repeatedly fertilized with Hakaphos special (COMPO GmbH, Münster, Germany).

Cuttings used for rooting experiments were excised from at least three months old stock plants. Rooting experiments were carried out as described previously [9]. At specific time points, 1 cm of the cutting base (rooting zone) was used for counting of root primordia and roots, immunological detection of AOC protein or was immediately frozen in liquid nitrogen and stored until use at −80 °C.

### Pharmacological experiments

Seedlings were germinated and grown under sterile conditions on ½ MS medium [33]. Roots of two weeks old seedlings were removed and de-rooted plantlets were transferred on Petri-dishes with ½ MS medium (supplemented with hormones as indicated) and cultivated vertically for 14 d under long-day lighting conditions at 22 °C (see Additional file 1: Figure S4). Root numbers of hormone-treated plantlets were determined in relation to plantlets grown on un-supplemented ½ MS medium.

### Generation of *PhAOC-RNAi* plants

A fragment of 180 bp was amplified from the *PhAOC* cDNA (GenBank: EU652410) using *Proofstart DNA-Polymerase* and the primers listed in Additional file 2: Table S3, cloned into *pENTR/SD/D-TOPO*-Vector (Gateway® Cloning, Invitrogen), and transferred into the RNAi vector *pHellsgate 8* [34]. Additionally the *ccdB* gene of *pHellsgate 8* was cut out to receive the empty vector control plasmid without the RNAi cassette (pHell). After transformation of *Agrobacterium tumefaciens* GV3101, leaf discs of *P. hybrida* cv. Mitchel were transformed with recombinant agrobacteria, and regenerated plantlets (T0 generation) were selected using polymerase chain reaction using primers listed in Additional file 2: Table S4 and as described previously [29, 35]. Two independent pHell and three independent *PhAOC-*RNAi lines (T1 generation) were selected from T0 plants for further analyses. From each transgenic line, eight plants were grown and used separately as ‘mother plants’ to generate cuttings for further analyses.

### Quantitative RT-PCR analysis

RNA isolation and determination of transcript accumulations of *PhAOC*, *PhACO1 (*SGN-U207414) *PhDevA-20-C01* (SGN-U212243) as well as *3CL9414* (*PhCWI)* by qRT-PCR were done as described by [9] using *Cytoplasmic ribosomal protein S13* of *P. hybrida* (*PhRSP13*; SGN-U207968) as reference gene. This gene was selected according to [36] and was tested in advance for even transcript levels in stem bases of cuttings (Additional file 1: Figure S5). Each reaction mix contained a 15 ng RNA equivalent of cDNA and 1 pm gene-specific primers. All assays were performed on at least eight biological replicates in three technical replicates each. Relative gene expressions were calculated by the comparative Cq method [37]. Real-time PCR primers were designed using Primer Express software (Applied Biosystems, Warrington, UK). Primer sequences are given in Additional file 2: Table S5.

### Extraction of proteins, immunoblot analysis, and assay of AOC activity

Proteins were extracted from 1 g of homogenized plant material with 50 mM sodium phosphate buffer, pH 7, containing 2 % PVPP and 0,05 % Tween 20 as described previously [38]. Resulting plants extracts were used for both, immunoblot analysis according to [38] as well as determination of AOC activity. The latter was performed according to [39] with the following modifications. Protein extracts containing 35 μg of total protein, recombinant *Hv*AOS activity (4 nkat; [40], and sodium phosphate buffer (50 mM; pH 7.0) were combined in a final volume of 200 μl. The reaction was initiated by the addition of 40 nmol 13(*S*)-HPOT. After incubation at 4 °C for 10 min, reaction was stopped by acidification. Me-OPDA was added as internal standard. Extraction with 2 ml of diethyl ether and evaporation of extract was performed followed by treatment with 0.2 M NaOH (in methanol) to activate *trans*-isomerization of *cis*-(+)-OPDA. After incubation at 4 °C for 60 min, reaction was stopped by neutralization with 2 N HCl. The reaction mixtures was extracted with 2 ml of diethyl ether, evaporated and subjected to HPLC using an *Eurospher100 C18 4 mm* column (Macherey-Nagel, Düren, Germany) and 75 % (*v/v*) solvent A (methanol) in solvent B (0.2 % acetic acid in H_2_O) at 1 ml/min. Fraction at R_t_ 12.0–13.4 min (OPDA) was subsequently separated isocratically in *cis*- and *trans*-isomers by HPLC with an *EC 200/4 Nucleotex beta-PM* column (Macherey-Nagel) and 65 % (*v/v*) solvent A in solvent B at 1 ml/min. The absolute content of OPDA was calculated using the internal standard. Percentage of enzymatic formed *cis*-OPDA was calculated according to [41].

### Immunocytochemistry

Immunocytochemical detection of AOC in stem bases was performed as described [42]. Small pieces of stems were fixed with 4 % (*w/v*) paraformaldehyde/0.1 % (*v/v*) Triton X-100 in phosphate buffered saline (135 mM, NaCl, 3 mM KCl, 1.5 mM KH_2_PO_4_, and 8 mM Na_2_HPO_4_) and embedded in polyethylene glycol 1500 (Merck, Darmstadt, Germany). Cross-sections of 5 μm thickness were used for immunolabeling with the rabbit polyclonal antibody raised against recombinant LeAOC [40] at a dilution of 1:1000. The use of pre-immune serum at the same dilutions served as a control and revealed no signals. As secondary antibody, goat anti-rabbit IgG conjugated with AlexaFluor488 (life technologies, Carlsbad, CA, USA) was used according to the manufacturer’s instructions. Sections were analyzed by confocal laser scanning microscopy using a LSM510 META (Carl Zeiss GmbH, Jena, Germany).

### Determination of primordia number

For counting the primordia number, 0.5 cm of each cutting base was fixed in a solution of 2 % (*v/v*) formaldehyde, 0,1 % (*v/v*) Triton X-100 in buffer (50 mM PIPES, 5 mM MgSO_4_, 5 mM EGTA, pH 6.9) at room temperature for two hours. Fixed stem segments were cut into 300 μm sections using a VT1000S microtome (Leica Instruments, Nussloch, Germany). Microscopic analyses were performed using an Axioplan microscope (Carl Zeiss).

### Determination of IAA, ACC, JA, JA-Ile and OPDA

About 0.5 g FW of homogenised plant material (stem bases of about 1 cm in length) pooled from at least three cuttings was extracted with 10 ml methanol. To quantify IAA and ACC, [^13^C_6_]-IAA and [^2^H_4_]-ACC, respectively, were added as internal standards in appropriate amounts before extraction. The homogenate was filtered and was placed on a column filled with 3 ml DEAE-Sephadex A25 (Amersham Pharmacia Biotech AB, Uppsala, Sweden). The column was washed with 3 ml methanol resulting after evaporation in extract E1. Subsequently, the column was washed with 3 ml of 0.1, 1.0, and 1.5 M acetic acid in methanol. The following elution with 4 × 3 ml of 3 M acetic acid in methanol resulted after evaporation in E2.

To determine ACC content, E1 was processed as described in [21]. Extract E2 was separated on preparative HPLC using method 1 as described by [43]. Fraction at R_t_ 10.5–11.9 min was evaporated, dissolved in 100 μl methanol and methylated with 200 μl ethereal diazomethane at 20 °C for 10 min. Afterwards the sample was evaporated, dissolved in 70 μl CH_3_CN and IAA content was determined by GC-MS as described by [43].

JA, JA-Ile and OPDA were determined as described by [43] using 1 g fresh weight of homogenized plant material per sample. For these determinations, leaves were mechanically wounded and harvested after 1 h.

All determinations were done at least from eight independent biological replicates, each represented by one plant, from which cuttings were excised.

### Determination of invertase activity and soluble sugar contents

Invertase activity was measured as described previously [44]. Determination of soluble sugar contents was performed photometrically by a coupled enzymatic assay as described by [44].

### Statistical analyses

Statistical analyses were performed using the software SPSS 17.0 (SPSS Inc., Chicago, IL, USA). All data sets were tested for normal distribution and homogeneity of variance using the Kolmogorov-Smirnov test and Levene test, respectively. One-way-ANOVA was followed by Tukey’s HSD test for significance.

## Additional files

Additional file 1: Figure S1. Genome structure of *PhAOC*. Figure S2. Accumulation of AOC protein in stem bases of cuttings of *Petunia hybrida* expressing *PhAOC*-RNAi. Figure S3. Accumulation of indole acetic acid (IAA) and *PhDevA-20-C01* transcripts in stem bases of *Petunia hybrida* cuttings. Figure S4. AR formation in de-rooted seedlings of *P. hybrida* treated with different concentrations of jasmonic acid. Figure S5. qRT-PCR analysis of putative reference genes used for transcript analyses in stem bases of *Petunia hybrida* cuttings. (DOCX 1376 kb) Additional file 2: Table S1. Number of ARs in de-rooted seedlings of *P. hybrida* wild-type treated with various concentrations of oxylipins, 2,4-D and ACC. Table S2. Number of ARs in de-rooted seedlings of *P. hybrida* wild-type non-treated or treated with jasmonic acid (JA) alone or in combination with 2,4-D or ACC. Table S3. Primer sequences for cloning of *35S*::*PhAOC*-*RNA*i into pENTR. Table S4. Primer sequences for selection of transgenic plants. Table S5. Primer sequences of *PhAOC, PhACO, Ph2-GH3, Ph3CL9414, PhRSP13* used in quantitative real-time PCR. (DOCX 26 kb)

## Abbreviations

ACC: Amino cyclopropane-1-carboxy acid
AOC: Allene oxide cyclase
AR: Adventitious roots
CWI: Cell wall invertase
JA: Jasmonic acid
JA-Ile: Jasmonoyl isoleucine
OPDA: *cis*-12-oxo-phytodienoic acid
2,4-D: 2,4-dichlorophenoxy acetic acid
