# Supplementary material for: Jasmonates act positively in adventitious root formation in petunia cuttings
Source: BMC Plant Biol. 2015 Sep 22;15:229. doi: 10.1186/s12870-015-0615-1 (PMC4579608; doi:10.1186/s12870-015-0615-1)
Supplement: Additional file 1: Figure S1. — Genome structure of PhAOC. Figure S2. Accumulation of AOC protein in stem bases of cuttings of Petunia hybrida expressing PhAOC-RNAi. Figure S3. Accumulation of indole acetic acid (IAA) and PhDevA-20-C01 transcripts in stem bases of Petunia hybrida cuttings. Figure S4. AR formation in de-rooted seedlings of P. hybrida treated with different concentrations of jasmonic acid. Figure S5. qRT-PCR analysis of putative reference genes used for transcript analyses in stem bases of Petunia hybrida cuttings. (DOCX 1376 kb) [file 12870_2015_615_MOESM1_ESM.docx]

**Additional file 1**

**Supplemental figures S1 – S5:**


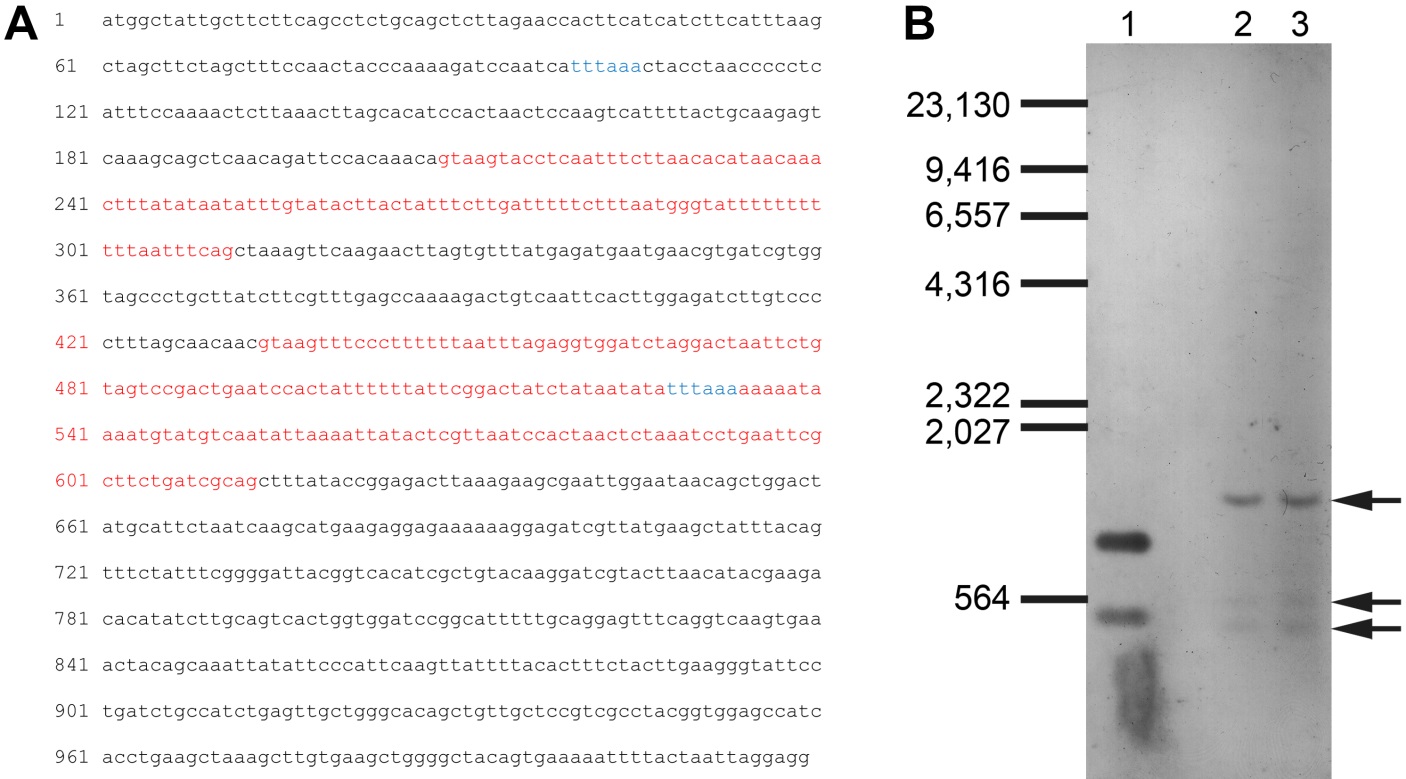


**Figure S1** **Genome structure of *PhAOC*.**

A. Genomic sequence of *PhAOC*. Exons and introns are given in black and red, respectively. Restrictions sites for *Dra*1 are highlighted in blue.

B. Southern blot analysis using *PhAOC*-cDNA (1) and genomic DNA from *P. hybrida* (2, 3) digested with *Dra*I and hybridized with DIG-labelled cDNA of *PhAOC*. Note that restriction of cDNA resulted in two hybridizing bands due to one restriction site within the cDNA and several sites within the vector backbone. The hybridization of *Dra*I-digested gDNA with *PhAOC*-cDNA shows three bands leading to the result that AOC of petunia is encoded by a single copy gene.





**Figure S2** **Accumulation of AOC protein in stem bases of cuttings of *Petunia hybrida* expressing *PhAOC*-RNAi.**

Stem bases of cuttings of wild type, two independent pHell lines (six plants each) and three independent transgenic lines transformed with PhAOC-RNAi (eight plants each) were collected one hour after excision. Protein extracts were separated on SDS-PAGE and processed for immunoblotting using a specific antibody binding to AOC. Coomassie-stained gel served as loading control (C).





**Figure S3** **Accumulation of indole acetic acid (IAA) and *PhDevA-20-C01* transcripts in stem bases of *Petunia hybrida* cuttings.**

Contents of IAA (bold line) is given as pmol g^−1^ FW; relative *PhDevA-20-C01* transcript accumulation (regular line) is shown as 2^-ΔCt^ values in relation to the reference gene *PhRSP13*. Each value is represented by the mean of three independent replicates ± SE. Hours post excision (hpe) of the cuttings is given on the *x*-axis.


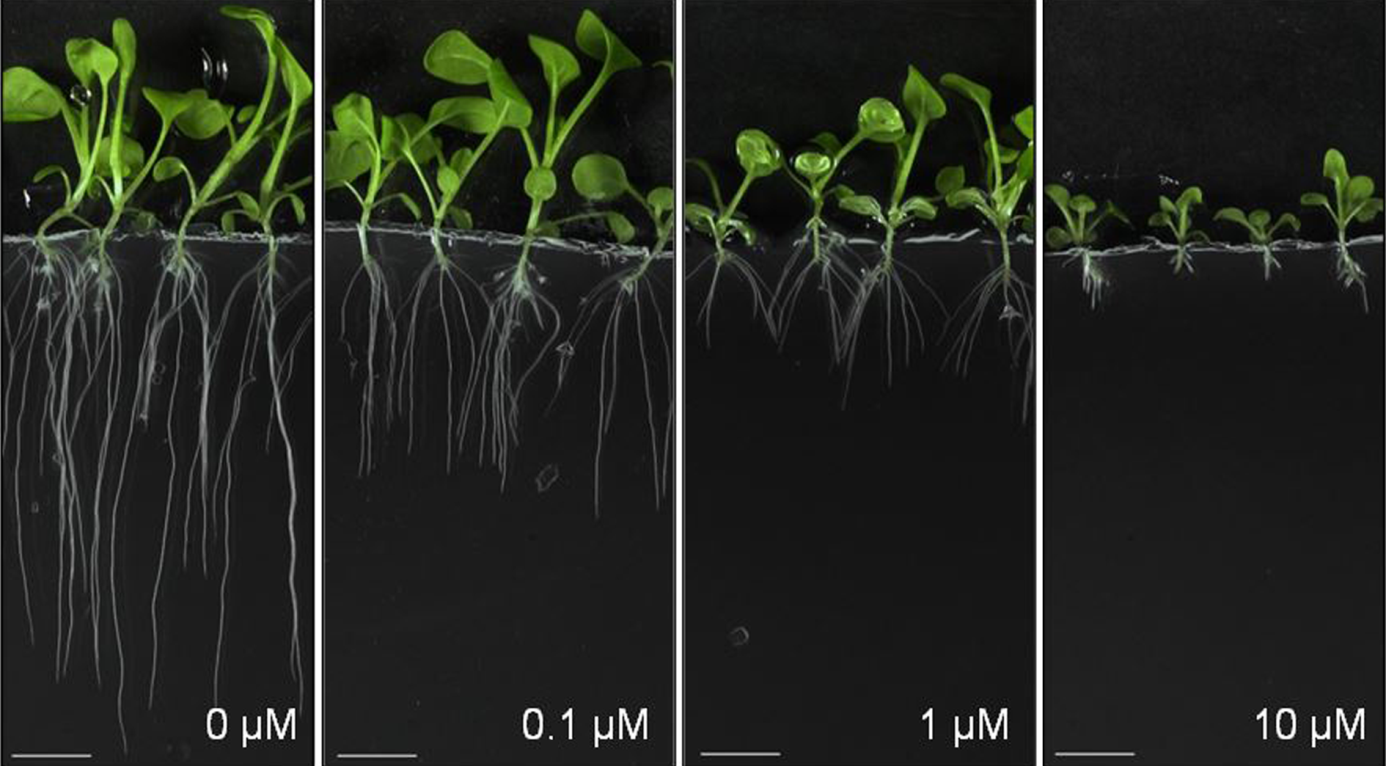


**Figure S4** **AR formation in de-rooted seedlings of *P. hybrida* treated with different concentrations of jasmonic acid.**

De-rooted seedlings transferred to medium supplemented with 0, 0.1 µM, 1 µM or 10 µM JA were grown for 14 days under long-day conditions. Increasing concentrations of JA reduced number and length of newly developed ARs. Bars represent 1 cm.


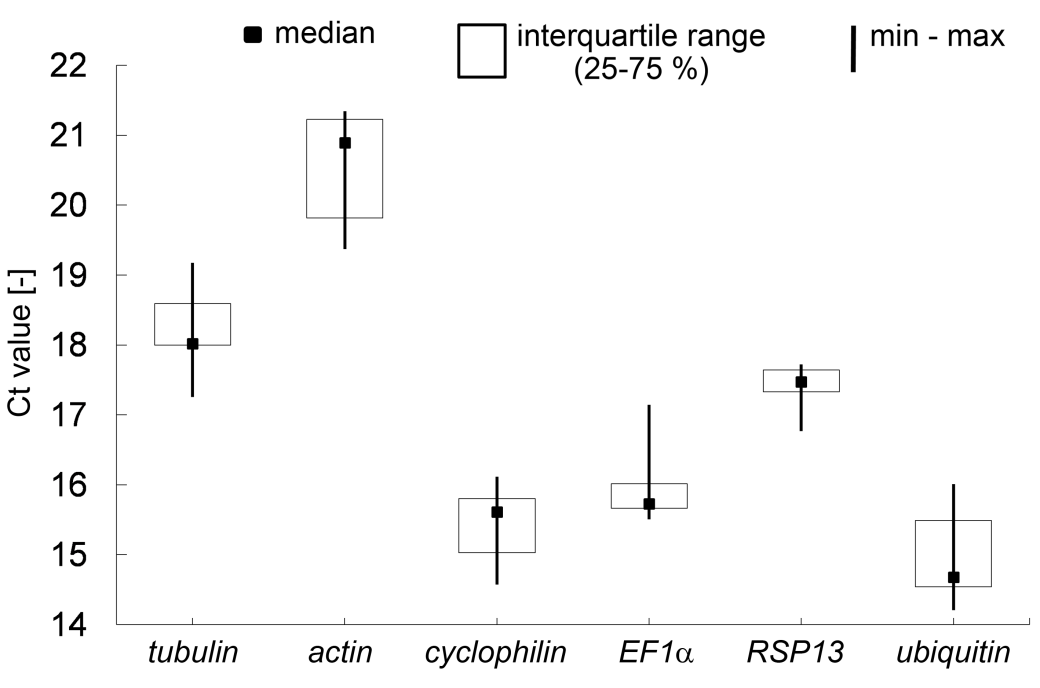


**Figure S5** **qRT-PCR analysis of putative reference genes used for transcript analyses in stem bases of *Petunia hybrida* cuttings.**

Stem bases of cuttings from *P. hybrida* were collected at 0, 0.5, 1, 2, 6, 12, 24, 48, 72, 120, 168, and 216 hpe. RNA isolated from stem bases of three cuttings each was used for determination of Ct values for *tubulin*, *actin*, *cyclophilin*, *EF1α*, *RSP13* and *ubiquitin*. Note that Ct values of *RSP13* show the smallest variation. Therefore, this gene served as reference gene in all further qRT-PCR experiments.
