# Supplementary material for: Jasmonates act positively in adventitious root formation in petunia cuttings
Source: BMC Plant Biol. 2015 Sep 22;15:229. doi: 10.1186/s12870-015-0615-1 (PMC4579608; doi:10.1186/s12870-015-0615-1)
Supplement: Additional file 2: Table S1. — Number of ARs in de-rooted seedlings of P. hybrida wild-type treated with various concentrations of oxylipins, 2,4-D and ACC. Table S2. Number of ARs in de-rooted seedlings of P. hybrida wild-type non-treated or treated with jasmonic acid (JA) alone or in combination with 2,4-D or ACC. Table S3. Primer sequences for cloning of 35S::PhAOC-RNAi into pENTR. Table S4. Primer sequences for selection of transgenic plants. Table S5. Primer sequences of PhAOC, PhACO, Ph2-GH3, Ph3CL9414, PhRSP13 used in quantitative real-time PCR. (DOCX 26 kb) [file 12870_2015_615_MOESM2_ESM.docx]

**Additional file 2**

**Supplemental Tables S1 – S5:**

**Table S1** Number of ARs in de-rooted seedlings of *P. hybrida* wild-type treated with various concentrations of oxylipins, 2,4-D and ACC.

| concentration (µM) | OPDA | JA | JA-Ile | 2,4-D | ACC |
| --- | --- | --- | --- | --- | --- |
| 0 | 12 ± 2^a^ | 12 ± 2^a^ | 12 ± 2^a^ | 12 ± 2^a^ | 12 ± 2^a^ |
| 0.1 | 10 ± 1^a^ | 12 ± 2^a^ | 11 ± 2^a^ | 13 ± 3^a^ | 10 ± 2^a^ |
| 0.5 | n.d. | n.d. | n.d. | 17 ± 5^b^ | n.d. |
| 1 | 10 ± 2^a^ | 10 ± 3^a^ | 11 ± 3^a^ | 19 ± 4^b^ | 8 ± 2^b^ |
| 10 | 9 ± 2^b^ | 7 ± 2^b^ | 9 ± 2^b^ | n.d. | 1 ± 2^c^ |
| 100 | 6 ± 2^c^ | n.d. | 4 ± 2^c^ | n.d. | n.d. |

Data of one out of three independent experiments are given as mean values ± SD (*n* = 10). Different letters within each column designate statistically different values (by one-way-ANOVA with Tukey’s HSD,P < 0.05).

n.d. = not determined.

**Table S2** Number of ARs in de-rooted seedlings of *P. hybrida* wild-type non-treated or treated with jasmonic acid (JA) alone or in combination with 2,4-D or ACC.

| Treatment | Number |  | Treatment | Number |
| --- | --- | --- | --- | --- |
| non-treated | 12 ± 2^ab^ |  | non-treated | 12 ± 2^ab^ |
| JA (0.5 µM) | 10 ± 2^ab^ |  | JA (1 µM) | 10 ± 2^ab^ |
| 2.4-D (0.1 µM) | 15 ± 4^ca^ |  | ACC (1 µM) | 15 ± 3^cc^ |
| JA + 2.4-D | 13 ± 3^bc^ |  | JA + ACC | 13 ± 2^bc^ |

Data of one out of three independent experiments are given as mean values ± SD (*n* = 10). Different letters within each column designate statistically different values (one-way-ANOVA with Tukey’s HSD test, *P <* 0.05).

**Table S3 Primer sequences for cloning of *35S*::*PhAOC*-*RNA*i into pENTR**

| **name** | **sequence (5`-3`)** |
| --- | --- |
| AOC-RNAi*-*for | CACCGGATTACGGTCACATCGCTGTACAAG |
| AOC-RNAi*-*rev | TGGCAGATCAGGAATACCCTTCAAGTAGAAAGTG |

**Table S4 Primer sequences for selection of transgenic plants**

| **transgene** | **name** | **sequence (5`-3`)** |
| --- | --- | --- |
| 35S::*AOC*-*RNA*i | Intron-for | TGTTAGAAATTCCAATCTGCTTGTA |
|  | Intron-rev | CGAACCCAATTTCCCAACTG |
| empty vector (pHell) | pHell-for | TATCCTTCGCAAGACCCTTC |
|  | pHell-rev | GGCGCTCTATCATAGATGTC |

**Table S5 Primer sequences of *PhAOC, PhACO1, PhDevA-20-CO1, 3CL9414 (PhCWI), PhRPS13* and other putative reference genes used in quantitative real-time PCR**

| **gene** | **sequence (5`-3`)** |
| --- | --- |
| *PhAOC*-for | CGGCATTTTTGCAGGAGTTT |
| *PhAOC*-rev | CCAGCAACTCAGATGGCAGAT |
| *PhACO1*-for | TACGTGCCCACACAGATGC |
| *PhACO1*-rev | GGGAGGAACATCGATCCATTG |
| *PhDevA-20-C01*-for | CCTATATTCCCATAATGGCCGTTGA |
| *PhDevA-20-C01*-rev | CATGTCCTCGATGAACTGAAGG |
| *3CL9414*-for | GTGGATGTTTGGCTTTTGTCC |
| *3CL9414*-rev | TTTCGTGACCCAACAACTGC |
| *RPS13*-for | AAGCTCCCACCTGTCTGGAAA |
| *RPS13*-rev | AACAGATTGCCGGAAGCCA |
| *actin*-for | TGCACTCCCACATGCTATCCT |
| *actin*-rev | TCAGCCGAAGTGGTGAAAGAG |
| *cyclophilin*-for | AGGCTCATCATTCCACCGTGT |
| *cyclophilin*-rev | TCATCTGCGAACTTAGCACCG |
| *EF1α*-for | CCTGGTCAAATTGGAAACGG |
| *EF1α*-rev | CAGATCGCCTGTCAATCTTGG |
| *tubulin*-for | TGGAAACTCAACCTCCATCCA |
| *tubulin-*rev | TTTCGTCCATTCCTTCACCTG |
| *ubiquitin*-for | TGGAGGATGGAAGGACTTTGG |
| *ubiquitin*-rev | CAGGACGACAACAAGCAACAG |
